# Nanocomposite scaffolds for accelerating chronic wound healing by enhancing angiogenesis

**DOI:** 10.1186/s12951-020-00755-7

**Published:** 2021-01-04

**Authors:** Hamed Nosrati, Reza Aramideh Khouy, Ali Nosrati, Mohammad Khodaei, Mehdi Banitalebi-Dehkordi, Korosh Ashrafi-Dehkordi, Samira Sanami, Zohreh Alizadeh

**Affiliations:** 1 Department of Tissue Engineering and Applied Cell Sciences, School of Advanced Technologies, Shahrekord University of Medical Sciences, Shahrekord, Iran; 2 Department of Virology, Iran University of Medical Sciences, Tehran, Iran; 3 School of Mechanical Engineering, College of Engineering, University of Tehran, Tehran, Iran; 4 Department of Materials Science and Engineering, Golpayegan University of Technology, Golpayegan, Iran; 5 Department of Molecular Medicine, School of Advanced Technologies, Shahrekord University of Medical Sciences, Shahrekord, Iran; 6 Department of Medical Biotechnology, School of Advanced Technologies, Shahrekord University of Medical Sciences, Shahrekord, Iran; 7 Endometrium and Endometriosis Research Center, Hamadan University of Medical Sciences, Hamadan, Iran; 8 Department of Anatomical Sciences, School of Medicine, Hamadan University of Medical Sciences, Hamadan, Iran

**Keywords:** Scaffold, Angiogenesis, Chronic wound, Wound healing, Skin tissue engineering, Nanobiotechnology

## Abstract

Skin is the body’s first barrier against external pathogens that maintains the homeostasis of the body. Any serious damage to the skin could have an impact on human health and quality of life. Tissue engineering aims to improve the quality of damaged tissue regeneration. One of the most effective treatments for skin tissue regeneration is to improve angiogenesis during the healing period. Over the last decade, there has been an impressive growth of new potential applications for nanobiomaterials in tissue engineering. Various approaches have been developed to improve the rate and quality of the healing process using angiogenic nanomaterials. In this review, we focused on molecular mechanisms and key factors in angiogenesis, the role of nanobiomaterials in angiogenesis, and scaffold-based tissue engineering approaches for accelerated wound healing based on improved angiogenesis.
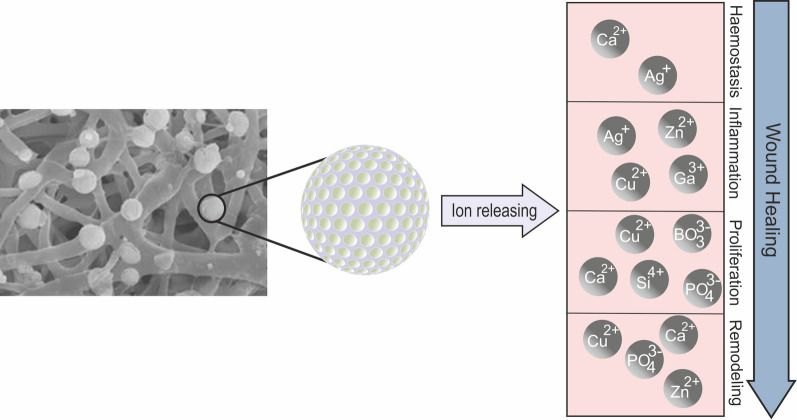

## Background

Wound healing is an accurate and well-orchestrated process in healthy individuals. Nevertheless, shortcomings in the wound healing lead to more than 38 million patients with chronic wounds worldwide, reaching epidemic proportions, causing a huge economic burden on the healthcare systems [1, 2]. It has actually been described that chronic wounds can have as profound an impact on the quality of life as renal and cardiovascular disorders. In addition, mortality nowadays rivals that of cancer patients for some patients with chronic wounds [3]. Autoimmune diseases, diabetes, aging, cardiovascular diseases, obesity, sensory neuropathies or constitute some of the reasons that cause the aforementioned pathology [4]. According to the latest Global Wound Care Market report, the sector has reached a value of about 20 billion dollars. Moreover, this amount is estimated to reach more than 25 billion dollars worldwide in 2023 [5]. It is therefore reasonable that adequate and well-planned management of chronic wounds has become important over the last decades in order to improve human quality of life and increase life expectancy.

In recent decades, various strategies have been developed for the treatment of chronic wounds. The most successful clinical strategy is skin autograft. Lack of immunogenicity is the most significant advantage of this method, but when the injured area exceeds more than 60% of the patient’s total body surface area, autografts could not cover the entire wounded site [6]. Pain, pigmentation disturbance and hair regeneration problems are other reported post-surgical issues related to the donor site [7]. Due to the limitations of this method, alternative strategies need to be developed to accelerate the process of wound healing. In order to develop novel approaches, a comprehensive understanding of the process of chronic wound healing and the mechanisms of the most important factors affecting the process are needed.

Wound healing is a complex multi-step process. In the first step, a clot plugs the wounded site. The repair and regeneration of the area would then continue with the formation of granulation tissue due to the introduction of fibroblasts, capillaries and immune cells into the clot. The edge of the wound would be aggregated and the epidermal layer would cover the surface of the wound. A balance between cell activity, such as proliferation, migration, differentiation and apoptosis, would lead to the creation of multilayer skin [8–10]. In this process, angiogenesis plays a significant role in improving the rate and quality of the healing process, and therefore the key role of angiogenesis is central to many studies of wound healing. In brief, angiogenesis implies the formation of new capillaries from pre-existing vessels to create a complex network of blood vessels [11]. Angiogenesis at the wound site provides more nutrition through blood flow and improves the healing process.

Many strategies to improve skin regeneration are based on stimulating and enhancing angiogenesis. In this regard, a promising approach is to use engineered structures, such as scaffolds with or without cells that can mimic the native tissue functionally. Such scaffolds could provide an appropriate microstructure similar to the extracellular matrix (ECM) for native cells to proliferate, migrate and differentiate. Various elements and factors, such as ions, nanoparticles and growth factors, could be incorporated in these structures to give them angiogenic properties. As an example, nanoparticles that are currently used as drug carriers could be incorporated into nanofibrous scaffolds that could provide a high surface/volume ratio for cell adhesion. The attached cells would be exposed to the angiogenic factors [12–15]. The use of nanocomposite scaffolds to improve angiogenesis in the field of skin tissue engineering has significantly increased over the last decade [16]. In this paper, we will discuss molecular mechanisms and key factors in angiogenesis, the role of nanobiomaterials in angiogenesis, and scaffold-based tissue engineering approaches for accelerated wound healing based on improved angiogenesis.

## Angiogenesis in wound healing

**Fig. 1:**
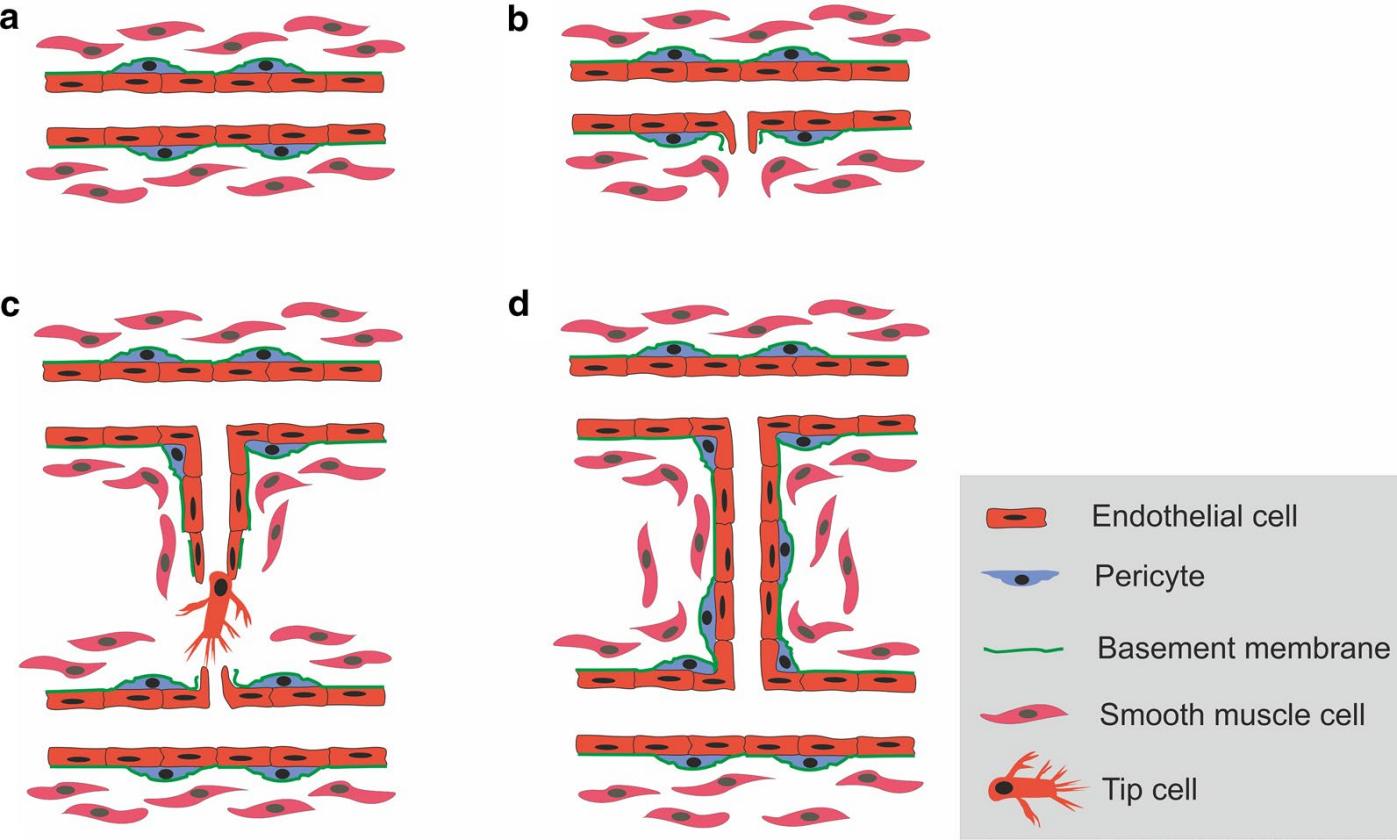
Main stages of angiogenesis [22–24]. A normal blood vessel (**a**). Angiogenic substances stimulate the angiogenesis process (**b**). Invasion of tip cells and lumen formation (**c**). Maturation of the new formed blood vessel (**d**)

Immediately after the accumulation of inflammatory cells following the injury, many angiogenic agents are secreted by these cells, causing the formation of new blood vessels. Defects in angiogenesis at this stage may lead to chronic wounds due to a slow rate of healing [11, 17]. After the inflammatory response following the injury, signals are transmitted to the endothelial cells that stimulate these cells to secrete matrix metalloproteinases (MMPs) and some other proteases leading to degradation of the basement membrane [18]. This situation provides an opportunity for the invasion of the tip cells into the surrounding matrix. The tip cells develop from the endothelial cells through the notch signaling pathway and are responsible for invasion and germination [19]. Proliferative stem cells are originated from other endothelial cells in order to develop vascular lumen. At last, the adherence of endothelial cells, with the help of vascular endothelial cadherin (VE-cadherin) as the main adhesive molecules, inhibits the proliferation to finalize vascularization [20]. Pericytes surrounded by basement membranes help to stabilize the recently formed vessels [21]. Figure 1 shows the main stages of angiogenesis process.

## Main factors of angiogenesis

### Vascular endothelial growth factor (VEGF)

Many studies have demonstrated that the VEGF family is the most important stimulating factor in the angiogenesis process. The main role of VEGF is to stabilize the vascular system through the formation of new networks of blood vessels. VEGF also involves the development of embryonic vascular system [25]. VEGF-A is the main stimulator of the blood vessels growth. VEGF-A is responsible for VEGFR2 phosphorylation, which induces endothelial cell migration, proliferation and differentiation [26]. Growth factors, cytokines, hypoxia and hormones are the most important regulatory factors for *VEGF*-*A* gene expression [27]. VEGFR1, VEGFR2 and VEGFR3 are the tyrosine kinase receptors of the VEGF family. VEGF-A, VEGF-B and PGF are related to VEGFR1. VEGF-A also has an affinity to VEGFR2. The primary receptor for VEGF-C and VEGF-D is VEGFR3 [28].

**Fig. 2:**
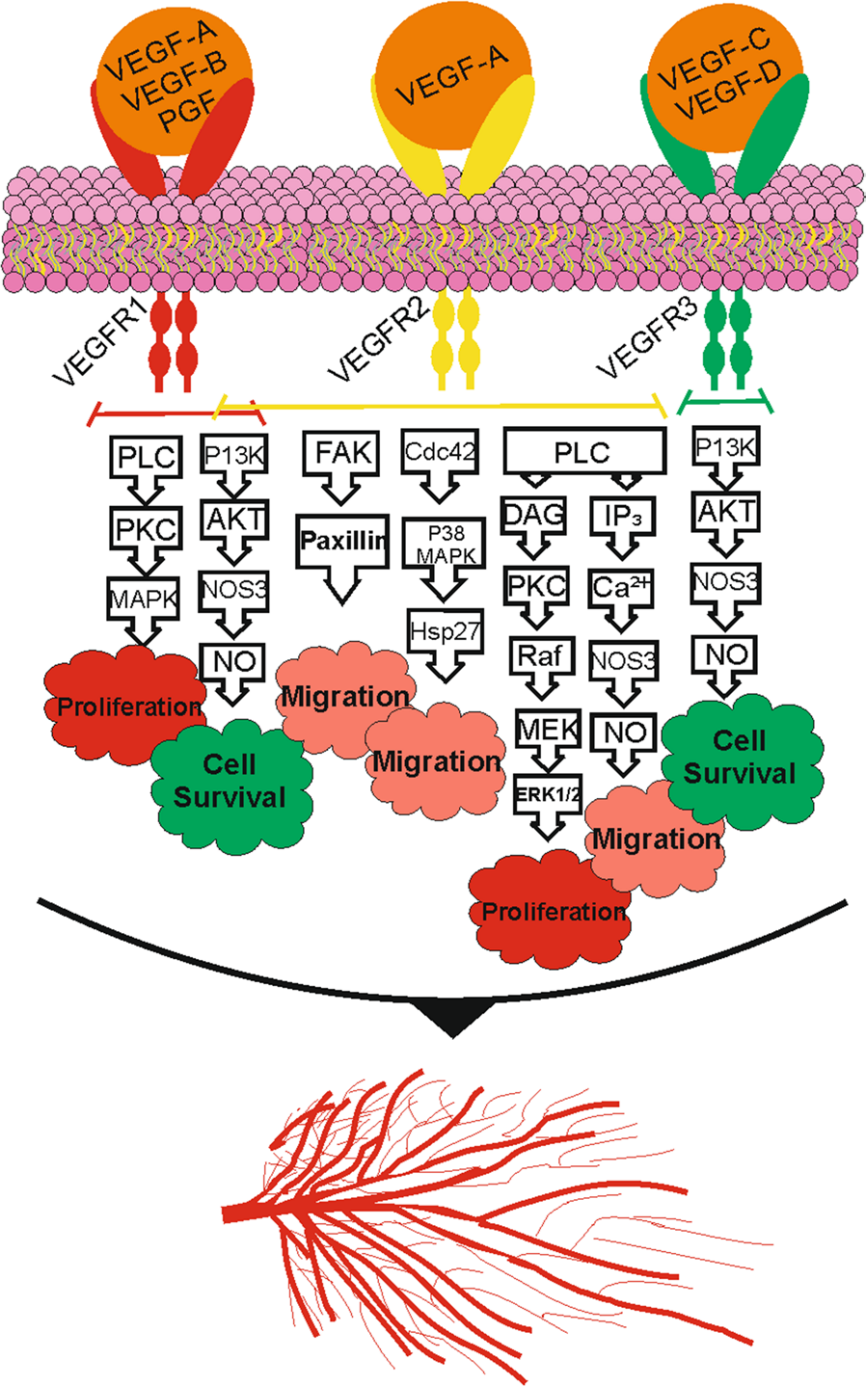
Signaling pathways induced by VEGF receptors following binding to their ligands [31–40]

VEGFR1 is structurally the same as VEGFR2. VEGFR1 is responsible for the negative regulation of VEGFR2 activities. VEGFR1 could transmit angiogenesis regulation signals by phosphorylation of downstream protein through an auto-phosphorylation process [29, 30]. VEGFR2 has a stronger tyrosine activity compared to VEGFR1, which can promote the survival, proliferation and migration of endothelial cells in order to improve angiogenesis [29]. One of the most important signaling pathways leading to endothelial cell proliferation is PLCγ/PKC/MAPK, which is induced by the binding of VEGF to VEGFR2 [31]. A summary of the signaling pathways induced by three types of VEGF receptors binding to their ligands is shown in Fig. 2.

### Angiopoietin

**Fig. 3:**
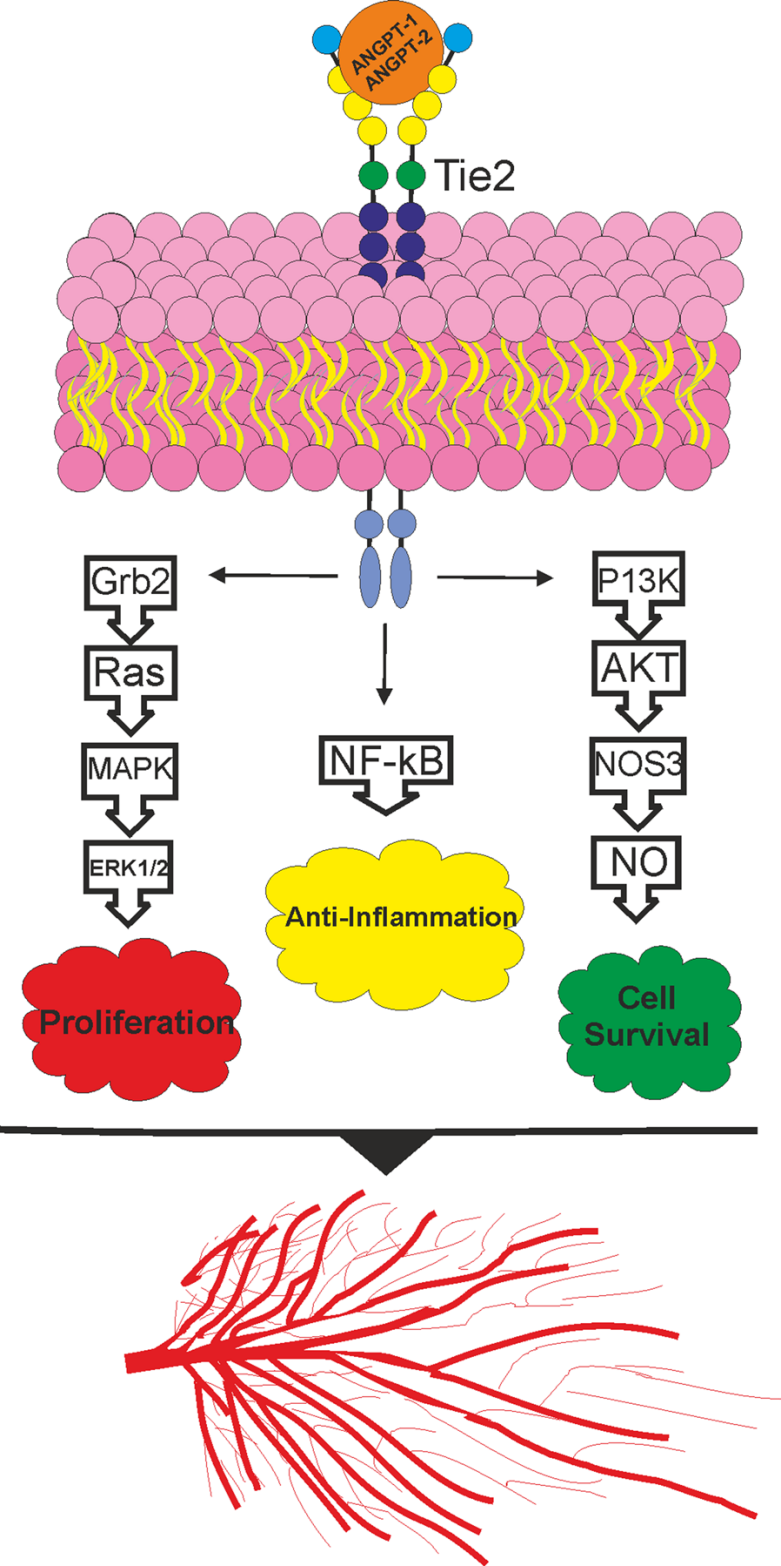
The role of ANGPT-1, ANGPT-2 and their receptor (Tie2) in angiogenesis [44, 45, 48–50]

Angiopoietin (ANGPT) plays a central role in the process of angiogenesis. It also regulates the permeability of vessels, causes vascular maturation, and keeps integrity and stability of the blood vessels. Survival and proliferation of endothelial cells and pericytes are affected by levels of ANGPT [41]. Receptors of ANGPT, Tie1 and Tie2, were identified in 1992. Tie2 is the receptor for ANGPT ligands and researchers have recently demonstrated the regulatory effects of Tie1 on Tie2 tyrosine kinase activity [42]. ANGPT-1 and ANGPT-2 were identified after the identification of their receptors. ANGPT-3 and ANGPT-4 were cloned from rodent and human sources, respectively [43]. Cells around the blood vessels are the primary source of ANGPT-1 expression. When ANGPT-1 is bound to its receptor (Tie2), the tyrosine kinase pathway is activated, leading to dimerization of the receptor by which tyrosine residues are phosphorylated. Cell viability, attachment, proliferation, migration and improved vessel stability are the results of studies obtained by activation of Akt and protein kinase B through the P13K signaling pathway [44, 45]. Yuan et al. showed the partial agonist/antagonist function of ANGPT-2 in Tie2 signaling in endothelium. ANGPT-2 also plays a key role in the regulation of angiogenesis by activating Tie2. In endothelial cells, the secretion of ANGPT-2 stimulated by growth factors, inhibits ANGPT-1/Tie2 signaling pathway (The function of ANGPT-1 is antagonistic). While, ANGPT-2 could bind to integrin to enhance angiogenesis [46, 47]. Figure 3 shows the role of ANGPT-1 and ANGPT-2 in angiogenesis.

### Fibroblast growth factors (FGFs)

Fibroblast growth factors (FGFs) are a large family of homologous peptides. Basic fibroblast growth factor (bFGF) is one of the most studied growth factors in cancer and angiogenesis studies [51]. As a result of its effects on endothelial cells and smooth muscle cells, bFGF induces angiogenesis as well as its function as a chemo-attractant and aids in the survival and proliferation of epithelial cells and fibroblasts [52].

Angiogenic properties of bFGF have been investigated by various authors in chick embryo chorioallantoic membranes (CAM) and rodent corneas [53, 54]. In addition to investigating the direct impact of bFGF on endothelial cell proliferation, Stavri et al. investigated the potential for bFGF to act indirectly through the upregulation of VEGF expression in vascular smooth muscle cells. They demonstrated that bFGF could also play an indirect role in stimulating angiogenesis by promoting VEGF expression [55]. An in vivo study conducted by Asahara et al. investigated the synergistic effect of VEGF and bFGF on the rabbit hind limb ischaemia model. The findings indicated a significant increase in angiogenesis when both growth factors were used together for treatment [56]. In 2007, Doi et al. produced a gelatin hydrogel-based controlled release system for bFGF. The effects of sustained release of bFGF on angiogenesis were evaluated in rabbits following removal of the femoral artery. bFGF treatment rabbits showed a concentration-dependent increase in vascular density, tissue perfusion and arteriole counts [57]. Recently, Yoo et al. have produced visible light-cured glycol chitosan hydrogels containing bFGF and EGF to accelerate the full-thickness wound healing process in Balb/C mice [58].

The following section discusses the common delivery systems used in chronic wound healing applications.

## Common approaches

Common approaches to improve angiogenesis are based on delivery systems (usually cells, proteins and growth factors) [59, 60]. The main issues of these systems are the short half-life of angiogenic factors and poor intake [61]. An alternative system for long-term induction of angiogenesis is the delivery of efficient genes to the injured tissue. The advantages of gene delivery systems are significant compared to protein delivery, which includes long-term expression and secretion of the angiogenic factor at the injured site, high efficacy, lower side effects, and increased activity [62, 63]. As an example, MicroRNA-135a-3p accelerates the process of angiogenesis by affecting the p38 signaling pathway in diabetic rodents, resulting in more effective wound healing [64]. Stimulating hormones, such as erythropoietin, are also used to improve the efficacy of the treatment [65]. Another approach is to use potent mesenchymal stem cells to enhance angiogenesis in the wound healing process [66].

Recent progress in nanobiotechnology has significantly improved potential applications in delivery systems. Nanobiomaterials exhibit appropriate physico-chemical properties to be used in medicine and in different fields of biology [67]. High surface/volume ratio, acceptable biocompatibility, loading efficiency and surface-modification capability are considered to be the most significant advantages of nanomaterials [68]. Based on these properties, a number of investigations have been conducted in the field of skin regeneration using nanomaterials in different ways. Some of these nanomaterials induce angiogenesis due to their unique physico-chemical characteristics [69, 70]. Bioglass nanoparticles (BG-NPs), some of metal-based nanoparticles, graphene-based nanomaterials and carbon nanotubes (CNTs) are examples of these angiogenic nanomaterials. Angiogenic nanomaterials could be incorporated into engineered structures such as scaffolds [71]. Scaffolds could be prepared from a wide range of natural or synthetic biomaterials. These biomaterials are usually biodegradable, which provide a slow release system for angiogenic nanomaterials to be released at an appropriate rate [72].

In the following sections, we will discuss appropriate characteristics of skin scaffolds, angiogenic nanomaterials and the applications of the incorporated scaffolds for wound healing and skin tissue engineering.

## Appropriate characteristics of a skin scaffold

### Mechanical properties

Scaffolds that are intended to be used for wound healing applications should have appropriate mechanical properties that can support cellular activities such as proliferation, migration and angiogenesis, as well as to protect structures found in native skin such as blood vessels, lymphatic systems and nerve bundles [73]. For this purpose, the skin scaffold should have mechanical properties similar to those found in native tissue. In this regard, the values of the tensile strengths, the Young’s modulus and the elongation-to-break are the most important parameters for assessing the suitability of the mechanical properties of the scaffold [74]. Although these values vary depending on the region of the native tissue, but according to the literature, the tensile strength between 5 and 40 MPa, the Young’s modulus between 4.5 and 25 MPa, and the elongation-to-break between 35 and 120% seem to be appropriate for wound dressings [75, 76]. Such values provide sufficient mechanical support for angiogenesis and tissue remodeling processes during wound healing and also prevent the side effects of stress shielding [77]. Many studies have shown the excellent mechanical properties of synthetic polymers such as polycaprolactone (PCL), polyurethane (PU) and poly (lactic-co-glycolic acid) (PLGA) due to their thermal and chemical stability [78, 79]. These polymers could also be blended with natural polymers such as chitosan, collage and gelatin, which have excellent biocompatibility in order to improve their mechanical properties [80–82].

### Porosity

In the field of skin tissue engineering, the porosity of a scaffold is critical to provide adequate spaces for cell accommodation, proliferation, migration and differentiation. Porous scaffolds also facilitate the oxygenation and nutrition of the wounded skin through the 3D matrix [83]. Some studies have shown that scaffolds with 60 to 90% porosity are suitable for wound healing applications as they are capable of providing the sufficient space for cell activity, oxygen and nutrient exchange, and the production of a new ECM [84]. Since the increase in the porosity of a scaffold has a direct effect on the reduction of the mechanical parameters mentioned above, the balance between the porosity and the mechanical properties of the scaffold is critical [85]. In order to prevent a significant reduction in the mechanical properties of the scaffold, nanobiomaterials such as carbon nanotubes and ceramic or metallic nanoparticles can be used which, in addition to increasing the mechanical properties, also stimulate angiogenesis [86, 87].

### Surface wettability of skin scaffolds

Wettability is one of the most significant features of the material’s surface. The surface hydrophilicity of skin scaffolds is a critical parameter that affects cell attachment, proliferation and differentiation [88]. The wettability of the scaffolds is usually determined by measuring the water contact angle at the surface of the scaffold [89]. According to other studies, moderate hydrophilic surfaces with a water contact angle between 30 and 70º have been shown to encourage cells to adhere and expand [90]. On the other hand, hydrophobic and highly hydrophobic surfaces exhibited lower cell adhesion. Presence of hydrophilic functional groups such as hydroxyl, esters and amides in the structure of many natural-based biopolymers make these biomaterials suitable to be used for skin tissue engineering applications [85]. Since the first step in angiogenesis is the attachment of cells to the scaffold, natural-based scaffolds incorporated with angiogenic nanobiomaterials are suitable for enhancing angiogenesis during the wound healing process. Hydrophilic wound dressings and skin scaffolds are also capable of providing moist environments that promote the healing process [91].

### Water vapor transmission rate and water uptake ability

As mentioned above, in addition to providing a moist environment, the ideal skin scaffold or wound dressing should also prevent dehydration of the wound and be able to remove the excessive wound exudate [92]. In this regard, an ideal skin scaffold should have a water absorption capacity of 100 to 800% (compared to its dry weight) to prevent the accumulation of fluids in order to enhance the formation of the new ECM [93, 94]. The accumulation of wound exudate at the injured site results in the degradation of ECM components and involvement of the surrounding tissues, which causes excessive pain to the patient [91]. In addition, it has been shown that skin scaffolds with water vapor transmission values ranging from 2000 to 2500 g/m2/day provide sufficient moisture and prevent the accumulation of wound exudate. Low water vapor transmission rates prevent gaseous exchanges leading to an accumulation of CO_2_ that can lead to the acidification of the wound media. This condition could have a direct impact on the regeneration of the injured tissue by inhibiting cell proliferation during angiogenesis. It is also provide an appropriate environment for the growth of anaerobic bacteria. On the other hand, high water vapor transmission rates can also lead to dehydration of the wound [91, 95].

### Degradation rate and release profile

In the case of biodegradable skin scaffolds, degradation rate and release profile are important parameters that should be considered. The higher degradation rate of the scaffold leads to the higher release rate of the incorporated bioactive factor [96]. On the other hand, the degradation rate of the skin scaffold should be proportional to the healing rate of the wounded skin [97]. Incorporation of nanobiomaterials can affect the physicochemical properties of the scaffolds and therefore their degradation rate and release profile [98]. Other important factors that can directly affect these two parameters are cross-linkers and crosslinking procedures. Chemical crosslinkers such as glutaraldehyde and *N*-ethyl-*N*′-(3-(dimethylamino)propyl) carbodiimide/*N*-hydroxysuccinimide (EDC/NHS) significantly reduce the rate of degradation of a wide range of biopolymers in both in vitro and in vivo aquatic environments. The cross-linking time and type of cross-linker should therefore be optimized according to the type of wound and the severity of the injury [99]. High levels of some angiogenic nanobiomaterials, such as metallic- and ceramic-based nanoparticles, could induce dose-dependent toxicity. The incorporation of high levels of these nanoparticles into scaffolds with an appropriate rate of degradation could provide a slow-release system that can stimulate angiogenesis without inducing dose-dependent toxicity [100, 101].

### Protein adsorption

When the skin scaffold is placed at the injured site, it is immediately exposed to proteins in the body’s fluids. The proteins are attached to the surface of the scaffold and provide an adhesive surface. Albumin is the most abundant protein in serum and after an injury occurs this protein is accumulated at the wound site. Subsequently, the absorbed albumin is replaced by fibronectin and vitronectin. The protein-coated surface could induce cell adhesion through membrane receptors [102]. As mentioned earlier, cell adhesion is the first step for angiogenesis. This could have a direct effect on skin scaffold biocompatibility [103]. Evaluation of albumin absorption can be an appropriate index to determine the ability of a skin scaffold or wound dressing to absorb proteins [85, 104].

Table 1 summarizes appropriate characteristics of a skin scaffold.

**Table 1:** Appropriate characteristics of a skin scaffold

	Characteristics	Value
Mechanical properties	Young’s modulus	4.5–25 MPa
	Tensile strength	5–40 MPa
	Elongation-to-break	35–120%
Physicochemical properties	Porosity	60–90%
	Surface wettability (water contact angle)	30–70º
	water uptake ability	100–800%
	Water vapor transmission rate	2000–2500 g/m^2^/day
	Degradation rate and release profile	N/A
Biological properties	Albumin adsorption	250–400 μg/mL/day
	Cell viability	N/A

## Angiogenic nanobiomaterials

### Metal nanoparticles

A wide range of metal-based nanoparticles have been widely used as angiogenic treatments for wound healing and skin regeneration. Zinc oxide nanoparticles (ZnO-NPs) have been shown to induce endothelial cell migration and enhance blood vessel formation by producing nitric oxide (NO) through the MAPK/Akt/eNOS pathway [105]. The incorporation of ZnO-NPs in scaffolds is a promising approach for skin tissue engineering applications. PCL/ZnO-NPs electrospun membrane was fabricated and characterized by Augustine et al. Increased proliferation of dermal fibroblast cells and upregulated expression of FGF2 and VEGF-A were reported. The results showed the potential of the electrospun membrane for vascular regeneration in wound healing [106].

The angiogenic properties of cerium oxide nanoparticles (CeO_2_-NPs) have been investigated in several studies. One of the most important investigations is a study by Das et al. They have demonstrated that CeO_2_-NPs stimulate pro-angiogenesis, depending on surface valance states. In vitro studies showed the formation of tube structures in the presence of CeO_2_-NPs. Vascular sprouting was the main result of in vivo studies to confirm the angiogenic activity of CeO_2_-NPs. The main causes of angiogenesis induction were stated to be stabilization of HIF-1α in endothelial cells and altered gene regulation [107]. The size of the CeO_2_-NPs and the ratio of Ce^3+^/Ce^4+^ are the parameters that could affect the angiogenic properties of the nanoparticles [108]. Another study demonstrated increased proliferation of endothelial cells, fibroblasts and keratinocytes in the presence of low concentrations of CeO_2_-NPs [109]. CeO_2_-NPs could be incorporated into polymeric scaffolds to be used as an angiogenic structure in tissue engineering. PCL/CeO_2_-NP nanocomposite membrane has been shown to be capable of promoting angiogenesis following subcutaneous implantation in rats due to the stability of HIF-1α and increased expression of VEGF [110].

Gold nanoparticles (Au-NPs) are commonly used in medical applications due to their unique physico-chemical properties. Au-NPs can scavenge reactive oxygen species (ROS) through antioxidant activity [111]. High surface-to-volume ratio of nanoparticles, make them more efficient. Using Au-NPs in photobiomodulation-based therapy accelerates the process of wound healing by improving angiogenesis as well as the proliferation of epithelial cells and the formation of collagen [112]. A study by Kim et al. showed that hydrocolloid membranes coated with chemically stabilized Au-NPs could be used for wound healing applications. Up-regulated expression of the ANGPT-1, ANGPT-2, VEGF and collagen genes confirmed the angiogenic properties of these stabilized nanoparticles [113]. Another approach is to use surface engineered Au-NPs as a delivery system to deliver angiogenic factors such as VEGF, as reported by Chen et al. [114].

### Bioactive glasses (BGs)

**Fig. 4:**
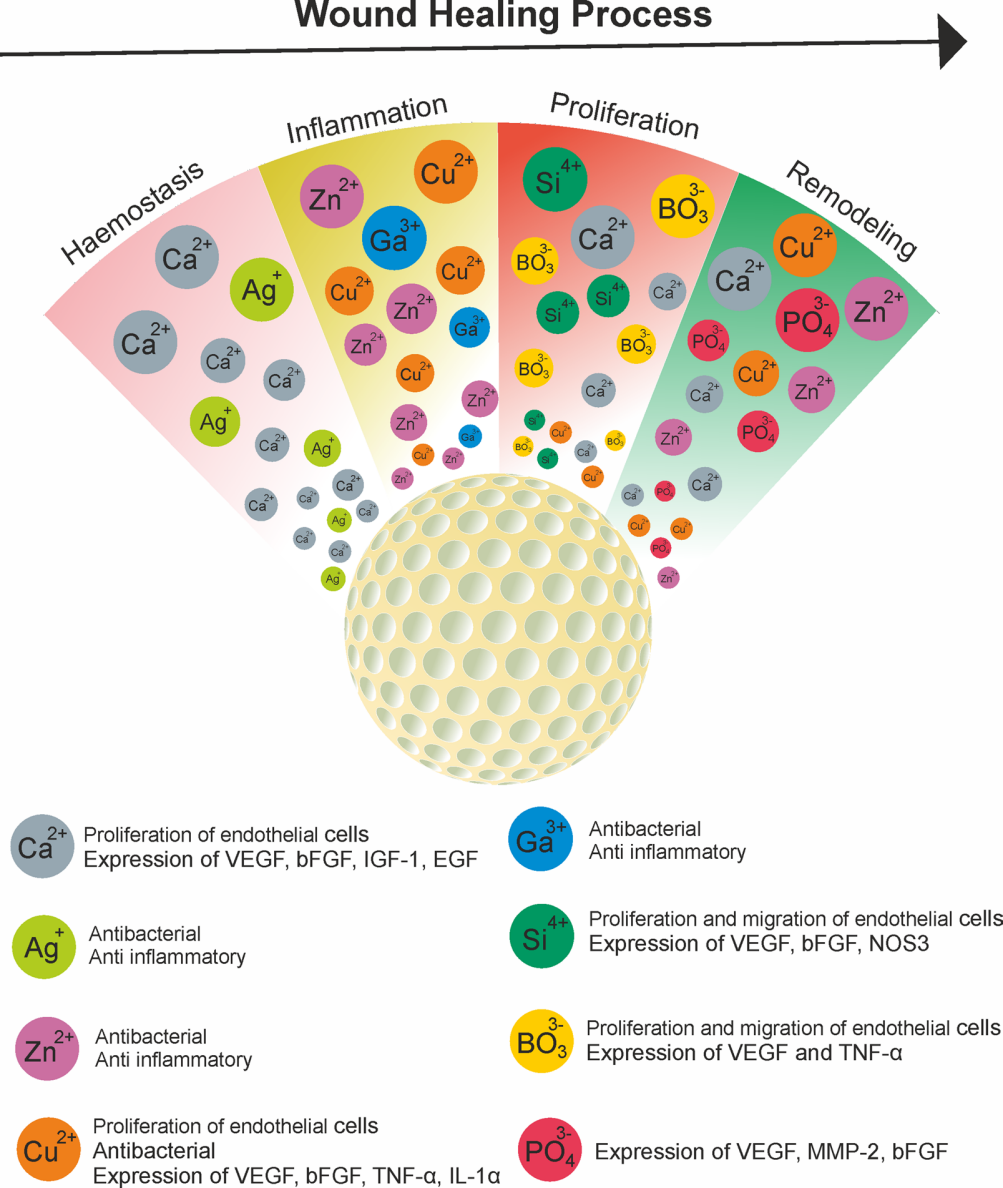
Ions release from incorporated mesoporous BG during the wound healing process [119–125]

BG is a series of specially designed silica-based glasses where the 3D SiO_2_ network is modified by the addition of CaO, Na_2_O and P_2_O_5_ [115]. BGs were first introduced in 1969 and described as a group of reactive biomaterials capable of bonding to mineralized bone tissue under physiological conditions. The first biomedical application of BGs was in the form of solid pieces for small bone replacements in middle ear surgery [116]. The most common BG is called 45S5, consisting of 45 wt % SiO_2_, 24.5 wt % CaO, 24.5 wt % Na_2_O, and 6.0 wt % P_2_O_5_. By mixing the different ratios of these four oxides, different BGs have been produced to enhance their inherent properties; additional oxides may also be used to improve specific therapeutic actions [115]. In recent years, BGs have been widely investigated for potential applications in the fields of regenerative medicine and tissue engineering due to their ability to increase angiogenesis and osteogenesis [117, 118]. BGs have attracted a great deal of interest, as their ion dissolution products have been identified to improve angiogenesis, which plays a key role in wound healing (Fig. 4).

BG-NPs have shown more advantages than conventional BGs, such as better biocompatibility, faster dissolution of ions, higher specific surface area, increased cell attachment, etc. BG-NPs are known as angiogenic nanobiomaterials and recent interest in BG-NPs increased in tissue engineering due to the dependence of accelerated wound healing on angiogenic materials [126, 127]. As the interest in BG-NPs increased, different methods of synthesis were developed. Gas phase (flame spray), micro-emulsion techniques, laser spinning, and sol–gel are the current BG–NP synthesis methods. The sol–gel method is the most common. BGs synthesized by the sol–gel method have an inherent mesoporous structure with a pore size of approximately 4–60 nm, which allows growth factors and drugs to be placed in nano-sized pores and then released locally in a controlled manner [128].

Some metal ions have been shown to increase angiogenesis by affecting key factors. The optional addition of these ions (e.g. Mg^2+^, Cu^2+^) to the BG-NPs could therefore promote the angiogenic properties of the particles and make them interesting to be used to accelerate the healing process (Table 2). In addition, ion-loaded BG-NPs could be incorporated into other polymeric biomaterials to improve their mechanical and biological properties. Such nanocomposite scaffolds have shown a good potential for wound healing and skin tissue engineering applications [87, 129].

**Table 2:** Angiogenic ions incorporated in BG-NPs [127, 132–141]

Angiogenic Element	BG Type	In vitro (cell type)/in vivo (animal model)	Results	Refs.
Calcium silicate	45S5 BG	In vitro (HUVECs)	Increased expression of VEGF, bFGF, KDR, bFGFR and NOS3 genes in HUVECs	[132]
Boron	45S5 BG	In vivo (CAM of quail embryos)	Increased expression of integrin α_v_β_3_, Increased number of blood vessels	[133]
Copper	Borate BG	In vitro (hBMSCs)/in vivo (rat with calvarial defects)	Increased in vitro proliferation of hBMSCs, Formation of new blood vessels was confirmed by IHC staining for CD31	[134]
Cobalt and Strontium	45S5 BG	In vitro (HUCPVCs)/in vivo (defect in the distal femur of rabbit animal model)	Increased expression level of VEGF gene in the HUCPVCs, formation of new blood vessels was confirmed by IHC staining for VEGF protein	[135]
Silicon	S53P4 BG	In vitro (Human CD‐18CO fibroblasts)	Increased proliferation of fibroblast cells, Increased secretion of VEGF, stimulation of neovascularization	[136]
Magnesium	Silicate BG	In vitro (HAECs)/in vivo (rabbit bone defect)	Increased proliferation of HAECs, HAECs alignment and exhibition of branch nodes that is a phenomenon of the primary stage of angiogenesis, Increased NOS3 gene expression, Promoted angiogenesis in the defect area	[137]
Europium	Mesoporous BG 45S5	In vitro (HUVECs)/in vivo (Mice with full-thickness wound)	Upregulated angiogenesis-related genes (MMP9, VEGFR1/2, CD31 and PDGFR α/β) of HUVECs, blood vessel formation, collagen deposition and re-epithelialization at chronic skin wound sites	[138]
Niobium	45S5 BG	In vitro (ST-2 bone marrow stromal cells)	Improved proliferation of bone marrow stromal cells, significant increase in VEGF release	[139]
Strontium	Borate BG	In vitro (hBMSCs)/in vivo (Critical-sized rabbit femoral condyle defect model)	Increased proliferation of hBMSCs, Upregulated expression of genes associated with angiogenesis and osteogenesis, such as VEGF, RUNX2, BMP-2, and osteopontin	[140]
Strontium and Copper	45S5 BG	In vitro (hBMSCs, HUVECS)	Differentiation of hBMSCs to vascular endothelial cells, formation of tubular and nodal networks of HUVECs	[127]

*HUVECs* human umblical vein endothelial cells, *CAM* chorioallantoic membrane, *hBMSCs* human bone marrow stem cells, *HUCPVCs* human umbilical cord perivascular cells, *HAECs* human amniotic epithelial cells

Biodegradable tiny cotton-candy borate BG (Mo-Sci Corp., Rolla, MO, USA), imitating the micro-structure of a fibrin clot, was reported to improve wound healing in both animals and human patients. These BG nanofibers (basic 13-93B_3_ composition: 53B_2_O_3_–6Na_2_O–12K_2_O–5MgO–20CaO–4P_2_O_5_ wt %), trade-named as DermaFuse™/Mirragen™, help impressively the healing of long-term venous stasis ulcers in diabetic patients, who were irresponsive conventional treatment [130]. Studies carried out in a rat subcutaneous model showed that the angiogenetic effect can be further accelerated by doping the BG with small amounts of copper that is locally released into the biological environment [131].

### Carbon nanotubes (CNTs)

CNTs are cylindrical molecules composed of single-layer carbon atom rolled-up sheets. CNTs have two single-walled and multi-walled forms. Single-walled carbon nanotubes (SWCNTs) have a diameter of approximately 1 nm or less. Multi-walled carbon nanotubes (MWCNTs) consist of several concentric nanotubes with a total diameter of more than 100 nm. The length of these structures is variable and may reach several micrometers or even millimeters depending on their use [142]. Nanostructure, large specific surface area, electrical conductivity and good mechanical properties make CNTs suitable for biomedical applications such as biosensors, drug and gene delivery systems, tissue engineering and regenerative medicine [86, 143, 144]. It has been shown that VEGF and matrix metalloprotein-9 (MMP-9), which are involved in angiogenesis and tissue remodeling, could be released by stimulated macrophages exposed to CNTs [139]. CNTs could be modified in order to be functionalized for angiogenesis regulation. Polyamine-coated CNTs were used as a micro-RNA delivery system to regulate angiogenesis by targeting endothelial cells [145]. In another study, Liu et al. treated the surface of CNTs with plasma. Then, VEGF was loaded onto the nanotubes to provide a new system with controlled release of VEGF. In the next step, porcine small intestinal submucosa was exposed to these modified VEGF-loaded CNTs. Results showed a statistically significant increase in angiogenesis during repair of the abdominal wall defect [146]. Using CNTs in the structure of polymeric scaffolds could also provide a suitable mechanical strength and give them biological advantages as studied by Lalwani et al. [147].

### Graphene-based nanomaterials

Graphene is a monolayer of carbon atoms bound in a honeycomb-like lattice with a two-dimensional sp^2^ structure. Such a unique structure is responsible for many specific properties of graphene, such as excellent mechanical properties, large surface area and exceptional thermal conductivity [148]. The potential of graphene was first explored for use in energy storage and sensors. The first biomedical use of graphene dates back to 2008 when it was introduced as a drug delivery carrier [149]. Since then, graphene has been considered as a potent biomaterial to be used in biomedical research. Biosensors, nanomedicine, delivery systems, imaging, diagnostics, regenerative medicine, biomedical and tissue engineering are the biological fields in which graphene-based materials are used [150–152].

Graphene oxide is one of the graphene-based materials with angiogenic properties. Graphene oxide is a unique graphene that is chemically modified and contains oxygen-containing functional groups (such as carboxylic acid, alcohol, hydroxyl, epoxide and carbonyl) in its structure with approximately three to one carbon/oxygen ratio. Over the last decade, graphene oxide has been widely used in biomedical applications [153, 154]. Angiogenic properties of graphene were investigated in a study conducted by Norahan et al., which demonstrated improved angiogenesis due to the use of a cardiac patch composed of collagen/graphene [155]. Appropriate concentrations of graphene oxide and its reduced form have been shown to enhance angiogenesis by induction of ROS production. Concentrations less than 100 ng have been reported to stimulate angiogenesis. Higher concentrations induce cell toxicity and inhibit angiogenesis [156, 157]. Qian et al. developed a 3D graphene oxide/PCL composite scaffold to regenerate injured sciatic nerves in vivo by promoting angiogenesis. Results confirmed upregulation of the PI3K/Akt/eNOS/VEGF signaling pathway compared to the control group (treated with PCL scaffold) which demonstrated the enhanced angiogenesis due to the presence of graphene oxide [158]. Nyambat et al. produced an adipose stem cell derived ECM-nano graphene oxide composite sponge for skin regeneration. The results showed the potential of the biocompatible angiogenic scaffold for skin tissue engineering applications [159].

### Europium hydroxide

**Fig. 5:**
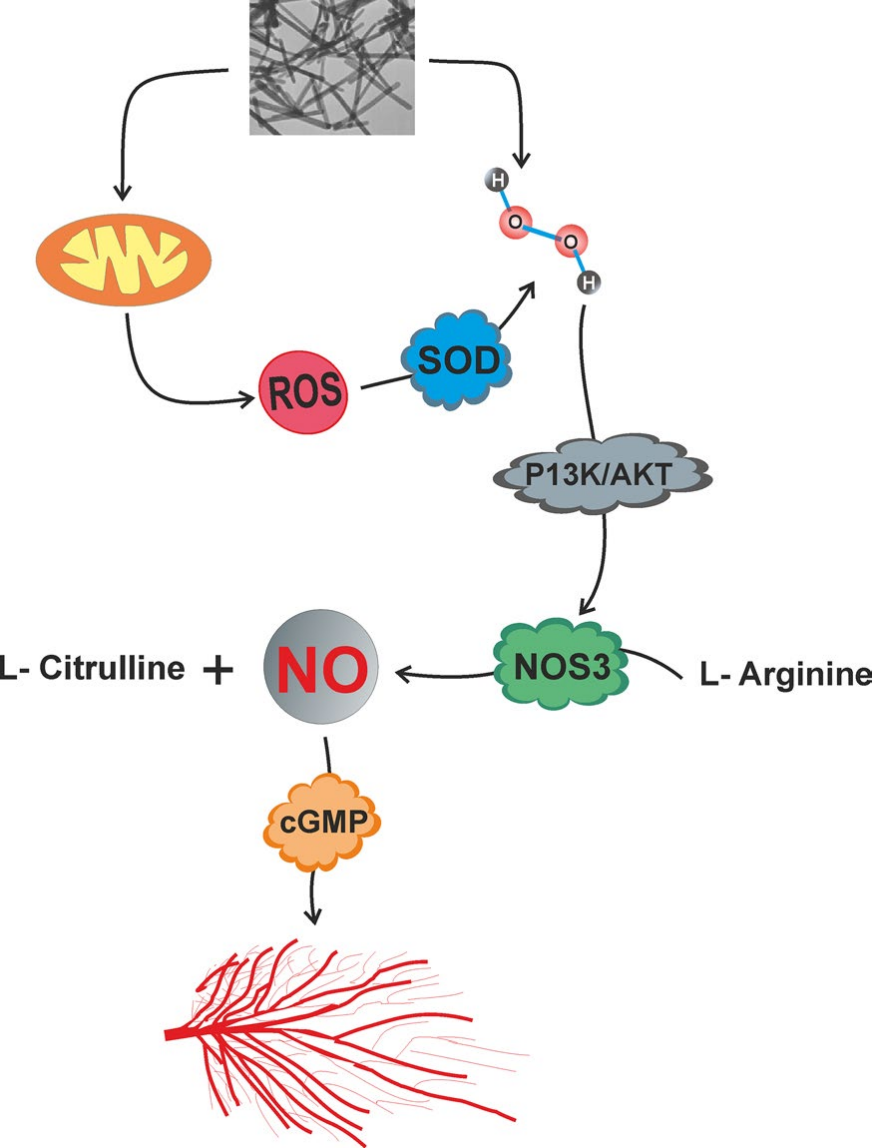
Mechanism of angiogenesis induced by europium hydroxide nanorods

Lanthanides are widely used elements in scientific research that have attracted researchers in immunoassay and biological imaging studies in recent years. Europium is the most reactive lanthanide that has been studied as a potent element to enhance angiogenesis in biological investigations in recent decades [160]. In 2008, Patra et al. studied the interaction between europium (III) hydroxide nanorods with human umbilical vein endothelial cells (HUVECs) to evaluate pro‐angiogenic properties of these nanorods. The results showed the internalization of the nanorods to the HUVECs. Chick chorio-allantoic membrane assay of HUVECS treated with the europium (III) hydroxide nanorods demonstrated vascular formation due to increased angiogenesis [161]. The molecular mechanism of angiogenesis induced by europium hydroxide nanorods was first suggested by Nethi et al. in 2015. Europium hydroxide nanorods has been shown to induce H_2_O_2_ production that activates the enzyme nitric oxide synthase 3 (NOS3) which has a protective function in the cardiovascular system. NOS3 triggers the PI3K/Akt signaling pathway to increase nitric oxide (NO) secretion, leading to angiogenesis [156]. The mechanism of angiogenesis induced by europium hydroxide nanorods is presented in Fig. 5.

The results obtained from such studies introduce europium oxide nanorods as a potent angiogenic material for use in tissue engineering applications. Europium oxide nanorods/PCL electrospun membrane has been shown to increase the rate of angiogenesis in chicken embryos compared to PCL electrospun membrane. The angiogenic activity was attributed to the ability of the scaffold to activate the VEGFR2/Akt signaling pathway due to the presence of europium oxide nanorods [162].

## Skin tissue engineering

New nanomaterial-based treatments for skin wounds have been developed in recent years. Several in vitro (studies conducted on endothelial cells such as HUVECs) and in vivo (rats, mice, rabbits and guinea pig models) studies have confirmed the efficacy of nanocomposite scaffolds on angiogenesis [16, 163]. Nanoparticles are widely used as an effective part of composite scaffolds to accelerate the healing process of chronic wounds through their various properties, such as angiogenic, anti-inflammatory and anti-bacterial properties [164]. Many clinical trials have been developed for skin regeneration based on nanoparticles, but Acticoat^®^ is the only US FDA approved nanoparticle-based skin product. The wound dressing contains silver nanoparticles that could be released slowly during the healing process. The antibacterial properties of these nanoparticles minimize the need for several wound dressings to be used during the healing of chronic wounds and also reduce the inflammatory response [165]. There is still no FDA-approved skin product with angiogenic nanomaterials in its structure, but the emerging number of related studies shows the potential of these nanobiomaterials for skin regeneration. The development of new techniques and methods facilitated the use of angiogenic factors, materials and nanoparticles in engineered structures and delivery systems [166]. However, a wide range of biomaterials have been characterized and used as composite and nanocomposite skin scaffolds, but there are many biological, engineering and clinical challenges that need to be considered. A summary of some of these challenges is presented in Table 3.

**Table 3:** Biological, engineering and clinical challenges in skin tissue engineering [167–170]

Biological Challenges	Engineering Challenges	Clinical Challenges
Selection of a suitable cell source	Selection of biocompatible material	Adaptation of the scaffold to the surrounding tissue
Repeatable cell differentiation condition	Optimal mechanical properties	Appropriate volume and shape of the regenerated tissue
Selection of growth factors, biomolecules and bio-active agents	Repeatable scaffold fabrication condition	Post-surgical nutrition and oxygenation

(Hydro-) gels are promising materials with great potential for development in the field of biology and medicine. They are widely used in wound healing and skin tissue engineering applications in a variety of ways [171]. (Hydro-) gel-based scaffolds usually exhibit appropriate biological properties despite their mechanical properties. The formation of (hydro-) gels with appropriate mechanical properties is challenging due to their natural low mechanical strength. The incorporation of nanomaterials is one of the feasible solutions for improving their stiffness. On the other hand, the structural network of (hydro-) gels provides an adhesive environment for nanostructures to present their biological properties more efficiently [172, 173]. The incorporation of nanostructures gives hydrogels specific properties that improve wound healing. Hydrogels incorporated with ZnO-NPs have been shown to support angiogenesis as studied by Ahtzaz et al. [174]. Adding graphene oxide-based nanomaterials to hydrogels can enhance cell attachment to improve angiogenesis in tissue engineering applications [175]. The incorporation of silicate BG-NPs into hydrogels supports the bonding of biomaterials and wounded tissues [87]. In a recent study by Li et al., silica-based nanocomposites hydrogel scaffolds were fabricated for enhancing angiogenesis to accelerate diabetic wound healing. Polyethylene glycol diacrylate (PEGDA) was used as the main network of the nanocomposite scaffold. Copper-containing BG-NPs and sodium alginate were added to induce angiogenesis. The in vivo results of the study showed increased number of vessels, high levels of blood flow volume, and accelerated wound healing (Fig. 6) [176].

**Fig. 6:**
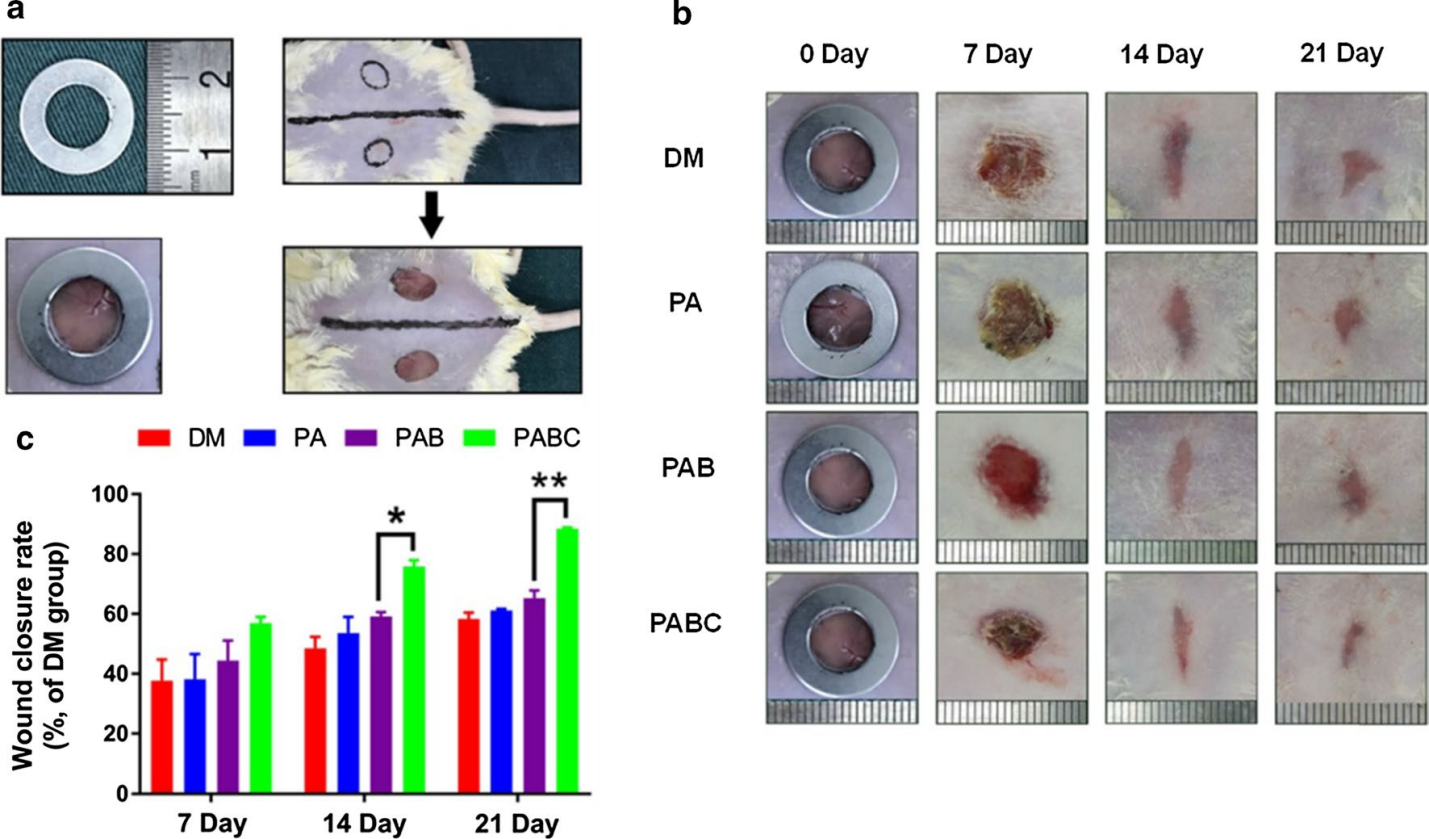
Effect of hydrogel scaffolds on diabetic wound healing. **a** Construction of diabetic wound model in ICR mice; **b** Wound healing process during 21 days treatment by different scaffolds (PA: PEGDA + sodium alginate, PAB: PEGDA + sodium alginate + BG-NPs, PABC: PEGDA + sodium alginate + copper-containing BG-NPs), DM: Diabetes mellitus wound was used as a control; **c** Wound closure rates at day 7, 14 and 21. (*p < 0.05 and **p < 0.01.). Reprinted with Permission from [176]

Incorporated (hydro-) gels are used in tissue engineering techniques to produce a wide range of skin scaffolds with angiogenic properties. Nowadays, three dimensional (3D) bio-printing of skin is a popular research area. Biomaterial selection is one of the key steps in this technique and the most common type of biomaterial used is biopolymer-based hydrogels. There is a wide range of natural, synthetic and composite biopolymers available for 3D skin bio-printing, which can be selected based on the type of printing process [177]. Both of the natural and synthetic biopolymers have certain limitations. Despite having the greatest advantage of imitating ECM, natural biopolymers exhibit poor mechanical properties. On the other hand, synthetic biopolymers have the advantage of excellent mechanical properties, but their microstructures are very different from the ECM of native tissue. Researchers therefore prefer to produce composite scaffolds, which have both natural and synthetic polymer components, in order to combine the advantages of both and overcome limitations [178]. Gelatin, chitosan, collagen and silk fibroin are the most common natural biopolymers used in printed skin scaffolds and the synthetic biopolymers are PCL, polylactic acid (PLA), and PLGA. Composite scaffolds are produced by a combination of at least one of each group [179–181].

Some basic requirements for bio-printing, summarized in Table 4, should be considered in order to achieve the optimal properties of skin scaffolds.

**Table 4:** Suitable properties of 3D bio-printing hydrogels [182–187]

Biocompatibility	Printability	Morphological properties	Mechanical properties	Physicochemical properties
Cell toxicity	Rheological properties and viscosity	Porosity percentage	Young’s modulus	Wettability
Blood toxicity	Sol–gel transition in response to temperature change	Pore size and shape	Strength	Degradation rate
Immunogenicity	Transition time	Micro-/nanostructure	Elasticity	Swelling
Protein adsorption	Shear thinning		Stiffness	Water vapor transition rate
Cell attachment			Elongation to break	

More than 200 polymers could produce nano- or micro-scale fibers using the electrospinning technique. All of these polymers are not proper for biological applications. Biocompatibility is the most important factor that determines whether a material is suitable for biomedical applications or not [188]. After identifying the biocompatibility of a polymer, other features that make a polymeric construct suitable for regeneration of a target tissue should be considered. Mechanical properties and degradation rate are important factors for producing an engineered skin scaffold [189]. Electrospun polymers provide a wide range of skin scaffolds for chronic wound healing applications. The most significant advantage of these types of nanofibrous scaffolds is the high surface/volume ratio that facilitates cell adhesion during the healing process. Biopolymers are used individually or in combination with other biopolymers [190]. Chitosan, alginate, chondroitin, collagen, gelatin, fibrin, keratin and silk fibroin are the most common natural polymers used single or blended to provide the electrospun scaffolds for skin tissue engineering [191, 192]. PCL, PLGA, poly (3-hydroxybutyrate-co-3-hydroxyvalerate) (PHBV), poly (etherurethane urea) (PEUU) and poly (glycerol sebacate) (PGS), are the examples of synthetic polymers used for regeneration of damaged skin tissue [169, 193]. The incorporation of nanostructures into these nano-/micro fibers is a common approach to providing controlled-release functional systems.

Porous polymeric sponges, in combination with nanobiomaterials and growth factor, can provide an appropriate microenvironment for cells homing, migration and differentiation. Porous scaffolds are prepared by different techniques such as freeze drying, particle leaching method and solid freeform fabrication. A wide range of natural and synthetic polymers are used to fabricate such porous scaffolds. Freeze drying is one of the most developed method to fabricate porous sponges. In this method, pores size could be controlled by changing amount of organic solvents and freezing temperature. Gas-foaming and solvent casting/particle leaching also give a suitable control over pores shapes and sizes. Incorporating of organic or inorganic fillers during scaffold preparation process is an efficient way to improve mechanical properties of composite scaffolds [194, 195].

Table 5 summarizes the application of engineered skin scaffolds incorporated with growth factors or nanobiomaterials to induce angiogenesis in wound healing.

**Table 5:** Different scaffolds with angiogenic properties for wound healing and skin tissue engineering applications

Biomaterial	Angiogenic factor/nanomaterial	Fabrication technique/method	Cross-linking	In vitro (cell type)/in vivo (animal model)	Results	Refs.
Collagen/Chitosan	VEGF-loaded PLGA microspheres	Freeze drying	–	In vitro (L929 mouse fibroblast)	Controlled release of VEGF; Proliferation of fibroblasts	[196]
Chitosan	SIKVAV peptide	Freeze dried hydrogel	–	In vivo (female C57BL/6 mice with full-thickness wound)	Re-epithelialization of wounds; Proliferation and differentiation of keratinocyte; inhibition of inflammation; Promotion of angiogenesis (increased expression of CD31)	[197]
Collagen/Hyaluronic acid	angiogenic growth factors (VEGF, PDGF, bFGF and EGF)	Electrospinning	EDC/NHS	In vitro (HUVECs)/In vivo (Male Sprague–Dawley diabetic rats)	Controlled release of angiogenic factors; Significant increase in HUVECs viability; Neo-vascularization (increased expression of CD31 and αSMA)	[198]
Hyaluronic Acid/Silk fibroin	ZnO-NPs	Electrospun Core–shell	–	In vitro (HaCat cells)/In vivo (rats with second-degree burn wounds)	Scaffolds with 3% ZnO-NPs significantly improved cell proliferation; Accelerate wound closure; Formation of new blood vessels	[199]
GelMA	Reduced Graphene Oxide	Freeze-dried hydrogel	UV radiation	In vitro (EA.hy926 endothelial cells, HaCat keratinocytes, and 3T3 fibroblasts)/In vivo (chicken embryo model)	No cell toxicity; Proliferation and migration of Cells; Promoted wound closure in scratch assay (wound healing assay); Increased angiogenesis in chicken embryo model	[78]
Chitosan/PEO	VEGF and PDGF-BB	Electrospinning	–	In vitro (HDFs)/In vivo (male Sprague–Dawley rats with full-thickness wound)	Promote the fibroblasts proliferation; Induction of angiogenesis; Epithelial regeneration; Collagen deposition and functional tissue remodeling	[200]
Silk fibroin/Sodium alginate	Strontium	Casting	–	In vitro (Mouse L929 fibroblasts)	Promote cell attachment and viability; Improving VEGF and bFGF secretion (induction of angiogenesis)	[201]
Gelatin/Sulfonated silk	basic fibroblast growth factor 2 (FGF-2)	3D printing	EDC-NHS	In vitro (primary child foreskin fibroblasts)/in vivo (male Sprague–Dawley rats with full-thickness wounds)	Increase in proliferation of fibroblasts; constant slow-release of FGF-2; Re-vascularization; Re-epithelialization; increased expression of α-SMA and CD31 on day 28 post-surgery	[202]
Chitosan-PEO/PCL-Collagen	bFGF, EGF and silver sulfadiazine	Electrospinning	–	In vitro (HDFs)/In vivo (male Sprague–Dawley rats)	Higher proliferation and attachment of fibroblasts; re-epithelialization; increased angiogenesis; decrease in inflammatory cells	[203]
Chitosan/PVA	NO	Freeze dried hydrogel	TEOS 2%	In vitro (HaCaT keratinocytes cells and 3T3 fibroblast cells)	Prolonged and sustained release of NO. Increased cell viability and proliferation	[204]
PCL	Y_2_O_3_-NPs	Electrospinning	–	In vitro (Mouse L-929 fibroblast)/In vivo (male Sprague–Dawley rats)	Proliferation of L-929 fibroblast; Increased expression of VEGF, EGFR (increased angiogenesis), downregulation of TNF-α, and COX-2 (Cycloxygenase-2) (decreased inflammation)	[79]
PCL	Europium hydroxide nanorods	Electrospinning	–	In vitro (HUVECs)	No aggregation of blood cells (RBC, WBC and platelets); enhanced adhesion, viability and proliferation of HUVECs; increased phosphorylation of Akt protein; increased expression of VEGFR2	[162]
PCL	ZnO-NPs	Electrospinning	–	In vitro (HDFs)/in vivo (guinea pigs with full-thickness skin wounds)	promoted proliferation HDFs on the PCL/ZnO-NPs scaffold; Increased expression of FGF2 and VEGF-A; Complete wound healing on 25^th^ day of study	[12, 106]
PCL	Titanium Nanorods	Electrospun mesh	–	In vitro (Mouse 3T3 fibroblasts and immortalized human HaCat Keratinocytes, HOECs), Scratch test, CAM Angiogenesis Assay/In vivo (Guinea Pigs, male Sprague–Dawley rats with full-thickness excision wounds)	Cell compatibility, adhesion and proliferation; Migration and proliferation of 3T3 cells and HaCat keratinocytes into the scratched area; Appearance of network of blood vessels growing around the scaffold Promote angiogenesis after subcutaneous implantation in Guinea pigs; Effective reduction in the wound size after 16 days in rats with full-thickness wounds	[78]
PHBV	CeO2-NPs	Electrospinning	–	In vitro (HOECs and HMECs); HaCat cells in scratch assay; CAM angiogenesis assay/In vivo (Male Sprague–Dawley diabetic rats with full thickness excision wounds)	Enhanced cell viability and adhesion of HOEC and HMEC; Migration of HaCat cells into the scratched area; Formation of blood vessels near the scaffold; Healing of full thickness excision wounds during 15 days of study	[205]
PCL/Gelatin	MgO	Electrospinning	–	In vitro (hEnSCs)/In vivo (male Wistar rats with full-thickness wounds)	Increased proliferation of hEnSCs; Promote wound area closure; increase in number of vascular structures	[206]
PLA-PVA	CTGF	Electrospun Core–Shell Membrane	–	In vitro (3T3 fibroblasts, HaCat Keratinocytes, EA.hy926 endothelial cells); In vitro wound healing assay (scratch); CAM assay	Higher fibroblast, keratinocyte and endothelial cell viability; Promote wound area closure in scratch test; Induction of angiogenesis in CAM model	[207]
PU-PDMS/Fibrin	PLGA nanoparticles loaded with VEGF and bFGF	Spray phase-inversion technique	–	In vivo (diabetic mice with full-thickness skin wounds)	accelerated wound closure at day 15 post-surgery; Complete re-epithelialization; Formation of new blood vessels	[208]
PVA/Chitosan/Gelatin	bFGF-loaded PCL microspheres	Freeze-dried hydrogels	–	In vitro (human fibroblast cells)/in vivo (male Wistar rats with full-thickness skin wounds)	Sustained release of bFGF; Adhesion and proliferation of human fibroblast cells on the surface of the hydrogel; Re-epithelialization, Enhanced angiogenesis after 20 days of treatment	[209]

*PLGA* poly (lactic-co-glycolic acid, *SIKVAV* Ser-Ile-Lys-Val-Ala-Val, *EDC/NHS* ethyl (dimethylaminopropyl) carbodiimide/*N*-hydroxysuccinimide, *HUVECs* human umbilical vein endothelial cell, *αSMA* α-smooth muscle actin, *GelMA* gelatin-methacryloyl, *PEO* poly (ethylene oxide), *HDFs* human dermal fibroblasts, *PCL* polycaprolactone, *EGF* epidermal growth factor, *PVA* poly (vinyl alcohol), *TEOS* tetraethoxysilane, *NO* nitric oxide, *COX*-*2* cycloxygenase-2, *RBC* red blood cell, *WBC* white blood cell, *HOECs* oral epithelial cells, *CAM* chorioallantoic membrane, *HMECs* human mammary epithelial cells, *MgO* magnesium oxide, *hEnSCs* human endometrial stem cells, *Y2O3*-*NPs* Yttrium oxide nanoparticle, *PHBV* poly (3-hydroxybutyrate-co-3-hydroxyvalerate), *CeO2*-*NPs* cerium oxide nanoparticle, *PLA* poly lactic acid, *PU* poly (ether)urethane, *PDMS* polydimethylsiloxane, *CTGF* connective-tissue growth factor

## Conclusion

Skin as an integral organ is the body’s first barrier against external pathogens. Any serious damage to the skin could have an impact on human health and quality of life. The management of the pathological conditions of this organ seems to be a critical issue in order to reduce the economic pressure on the medical system and patients. In recent decades, different approaches have been developed to improve the rate and quality of chronic wound healing. What is evident is the essential role of angiogenesis in the regeneration of damaged skin. Common approaches for improving angiogenesis are based on cell, protein and gene delivery systems. Recent progress in nanobiotechnology has significantly improved potential applications in delivery systems. Developed methods in material science and tissue engineering provide an opportunity to use angiogenic nanobiomaterials in the design of engineering structures to provide complex, sustained release systems. In this paper, we reviewed the different uses of angiogenic nanobiomaterials in chronic wound healing and skin tissue engineering applications based on previous studies.

As mentioned above, the generation of a functional vascular network is one of the major challenges in the regeneration of damaged skin. For this purpose, the quality of the regenerated vascular network is more important than quantity. In other words, functional blood perfusion over the vascular network is the criterion, not just the number of regenerated vessels. The importance of the organization and maturation of the vascular structure is therefore clear. On the other hand, over-stimulating angiogenesis leads to the generation of many unorganized vessels with poor blood perfusion and inefficient performance. The organization of vascular structures is not only a factor that determines the quality of the regenerated tissue, but it seems to be a basic principle. Incorporation of bioactive molecules and nanobiomaterials with angiogenic properties is a growing strategy that is highly useful for improving tissue regeneration by improving angiogenesis and vascularization, but achieving tissue with normal and functional vascular structures remains a challenge.

## Data Availability

Not applicable

## Abbreviations

HUVECs: Human umblical vein endothelial cells
CAM: Chorioallantoic membrane
hBMSCs: Human bone marrow stem cells
HUCPVCs: Human umbilical cord perivascular cells
HAECs: Human amniotic epithelial cells
PLGA: Poly (lactic-co-glycolic acid)
SIKVAV: Ser-Ile-Lys-Val-Ala-Val
EDC/NHS: *N*-ethyl-*N*′- (3- (dimethylamino)propyl) carbodiimide/N-hydroxysuccinimide
αSMA: α-Smooth muscle actin
GelMA: Gelatin-methacryloyl
PEO: Poly (ethylene oxide)
HDFs: Human dermal fibroblasts
PCL: Polycaprolactone
EGF: Epidermal growth factor
PVA: Poly (vinyl alcohol)
TEOS: Tetraethoxysilane
NO: Nitric oxide
COX-2: Cycloxygenase-2
RBC: Red blood cell
WBC: White blood cell
HOECs: Oral epithelial cells
CAM: Chorioallantoic membrane
HMECs: Human mammary epithelial cells
MgO: Magnesium oxide
hEnSCs: Human endometrial stem cells
Y2O3-NPs: Yttrium oxide nanoparticle
PHBV: Poly (3-hydroxybutyrate-co-3-hydroxyvalerate)
CeO2-NPs: Cerium oxide nanoparticle
PLA: Poly lactic acid
PU: Poly (ether)urethane
PDMS: Polydimethylsiloxane
CTGF: Connective-tissue growth factor
